# From problem solving to problem definition: scrutinizing the complex nature of clinical practice

**DOI:** 10.1007/s40037-016-0314-0

**Published:** 2016-12-05

**Authors:** Sayra Cristancho, Lorelei Lingard, Glenn Regehr

**Affiliations:** 1 Centre for Education Research & Innovation, Schulich School of Medicine and Dentistry, Western University, London, Ontario, Canada; 2 Centre for Health Education Scholarship, Faculty of Medicine, University of British Columbia, Vancouver, Canada

**Keywords:** Perspectives, Interactions, Systems Engineering

## Abstract

In medical education, we have tended to present problems as being singular, stable, and solvable. Problem solving has, therefore, drawn much of medical education researchers’ attention. This focus has been important but it is limited in terms of preparing clinicians to deal with the complexity of the 21st century healthcare system in which they will provide team-based care for patients with complex medical illness. In this paper, we use the Soft Systems Engineering principles to introduce the idea that in complex, team-based situations, problems usually involve divergent views and evolve with multiple solution iterations. As such we need to shift the conversation from (1) problem solving to problem definition, and (2) from a problem definition derived exclusively at the level of the individual to a definition derived at the level of the situation in which the problem is manifested. Embracing such a focus on problem definition will enable us to advocate for novel educational practices that will equip trainees to effectively manage the problems they will encounter in complex, team-based healthcare.

Problem solving has been one of the central areas of exploration for researchers studying medical expertise. This work has revealed some important insights into how medical experts grapple with addressing relatively stable, well-defined problems. However, in focusing on problems that are singular, stable, and solvable, these insights might have limited value in preparing clinicians to deal with the complexity of the 21st century healthcare system in which they will provide team-based care for patients with complex medical illness [1]. In the medical education literature, this concern is gaining recent attention as researchers begin to challenge taken-for-granted assumptions about clinical reasoning activities, such as diagnosis [2]. When activities such as diagnosis or treatment planning are framed as ongoing processes of meaning making, problem solving can no longer be thought of merely as converging on the correct solution. Consider the following example, derived from a research interview with a senior general surgeon:

Dr. Smith was scheduled to operate on a gentleman with a pelvic exoneration that required removing all the organs within the pelvis. Chemotherapy prior to surgery would likely increase the success of surgery. The patient was assessed by the oncologist who decided not to provide chemotherapy before the operation because the oncologist thought the patient was *‘not reliable’*. To determine what to do, Dr. Smith engaged in a series of conversations with the nurse, the social worker, the oncologist and a more senior colleague. Dr. Smith felt very strongly that *‘treating someone with less than the standard of care’* was inappropriate. Yet, the oncologist continued to resist. As the day of the surgery approached, Dr. Smith gathered support from the rest of the team members involved and essentially ‘*tricked the oncologist’* by admitting the patient to the hospital in advance. With this decision, Dr. Smith presented a new situation to the oncologist saying *‘ok, well, now he’s in hospital, he’s very reliable; he’s not going anywhere … now you can treat him’*. The oncologist reframed his concern, remarking back to the surgeon that *‘sometimes what is good for this patient may not necessarily be good for society’*. Notwithstanding this stance, the oncologist acknowledged that since the patient was already in the hospital, he had to fulfil his professional responsibility of providing chemotherapy. As this treatment was taking place, Dr. Smith felt *‘somewhat morally conflicted because I get what the oncologist is worried about …’. *He now reported viewing the situation as *‘teetering’* between doing the right thing for one patient and imposing a significant cost on the system. In describing the shifting context that defined his efforts to help this patient, he characterized the situation as being one of making judgments like a rock climber: *‘very, very slow, inching your way … that feels like a solid hand-hold, ok, I’ll take that … or that doesn’t seem like it’s gonna go, so maybe I’ll try a different strategy, and often what you do at a certain moment in time, opens up what you can see at another moment … I think it’s like being in a constant evolution of finding your place.’*

What does this story reveal about the nature of problem solving in every day clinical practice? First, there was not a single problem in play here, but rather a constellation of problems defined differently from different perspectives. For example, from the surgeon’s perspective the question of whether the patient should receive chemotherapy was an issue of pursuing optimal care for a successful outcome, whereas from the oncologist‘s perspective, it was an issue of whether the treatment could be successfully enacted given the patient’s unreliability. Second, in the eyes of the surgeon (and the oncologist), the problem was not stable, but rather constantly evolving over time. Dr. Smith started with a procedural problem (operating on a patient that required removing all organs within the pelvis and identifying chemotherapy as needed to maximize success), which shifted into a care access problem (convincing the oncologist that treating the patient with chemotherapy prior to surgery was the most appropriate action), then became a team dynamics problem (convincing others of the need to admit the patient and *tricking* the oncologist). Finally, it evolved into a moral problem (the dilemma of doing the right thing for the patient vs. the cost imposed on the system), one imposed by the oncologist’s efforts to respond to his own shifting definition of the problem. Thus, what on the surface appears to be a single central problem of care for the patient is in fact a constantly evolving constellation of problems: it looks different depending on whose point of view we take, and on which point in time we emphasize during the process. The complexity in this team interaction is, therefore, not only, or even primarily, one of problem solving – it is also one of problem defining.

This story of everyday, incremental problem solving and iterative problem definition is the sort that occurs regularly in healthcare, and it illustrates the fluidity of problems. In Dr. Smith’s story the issue of reframing problems was made explicit as he told the story during his interview, but in everyday practice most likely it happens largely tacitly as clinicians balance their various priorities [3]. Whether explicit or tacit, however, team members learn from such experiences. The next time this senior surgeon and oncologist interact regarding pre-surgical chemotherapy, each will make inferences about the other’s definition of the clinical problem based on this experience and adapt their behaviour accordingly. The surgeon might, for example, admit the patient before consulting with the oncologist, both in an attempt to manage the problem definition and its solution. Thus, through experience, clinicians become savvy definers of problems just as they become savvy solvers of problems. Yet such sophisticated considerations are seldom articulated, and when the pre-emptive solution is enacted the problem does not manifest. Thus, the complexity and multifaceted nature of the underlying problem definition is hidden (perhaps, over repeated enactments, even from the physician himself).

Such examples challenge the conventional premise that problems are singular and stable, and that they can be ‘solved’ once and for all. A singular, stable definition and permanent solution of problems may hold in simple healthcare situations (e. g., a child with a minor ear infection brought to the family physician), but in complex, team-based situations like the one above, these premises are regularly challenged. Because different stakeholders may well approach the same issue differently, the major challenge lies not just in agreeing how to solve the problem, but in appreciating exactly what the problem is at any given moment in time.

If we acknowledge that clinical problem definition is not simple and straightforward, but multifaceted, evolving and iterative, then we require a new language for talking about *problem solving*. A number of other disciplines have grappled with this issue [4], and one that has developed a useful language is SSE [5–8]. Elsewhere we have discussed the implications of SSE in relation to other domains such as resilience [9] and learning [10]. For the purposes of this paper, we are exploring the value of SSE for reconsidering the construction of problem solving in the medical education literature. One of many concepts that Soft Systems Engineering posits [11–13] is the idea that because real-life problems usually involve divergent views and evolve with multiple solution iterations, the focus needs to shift in two ways. First, it needs to shift from *problem solving* to *problem definition*. Second, it needs to shift from a problem definition derived exclusively at the level of the individual to a definition derived at the level of the situation or system in which the problem is manifested [11].

SSE offers a vocabulary for making these shifts possible. First, SSE identifies the *perspectives* that are involved in a problem situation: who are the stakeholders in the problem and from what orientation does each define it? In the story above, different team members held different and conflicting perspectives on how to treat this particular patient prior to surgery. While the oncologist struggled with the idea of providing chemotherapy prior to the operation, the surgeon struggled with the consequences of admitting the patient earlier to the hospital. Second, SSE moves beyond a consideration of each isolated perspective to examine the *interactions* among them [11–13]. Although we might potentially interpret these differing perspectives as just a set of individual problems, in fact, these interacting perspectives collectively shape and reshape the situation in which each individual is enacting his or her part [14, 15].

In the surgery story, the interactions among the surgeon’s and the oncologist’s perspectives resulted in a series of redefinitions of the problem for the surgeon: from attending to the procedural steps, to attending to the team dynamics, to attending to the moral dilemma. Thus, under the SSE approach, the construction of the situation becomes a cyclical process: the dynamic nature of the various *perspectives* a single team member holds and the *interactions* with other team members’ perspectives reshapes the situation, which in turn influences every team member’s understanding. As a research approach, SSE therefore enables us to ask questions such as:

- What are all the relevant perspectives for the definition of a problem? In the story above these included both the surgeon and the oncologist, but might also have included those of the nurses, social workers, hospital administrators, and patient. As the surgeon told it, these perspectives seemed to align with his, but it would be important to ask as an open question.
- How do those perspectives interact with one another to iteratively define the problem? The surgery story showed at least three redefinitions of the problem caused by the interactions among perspectives.
- How do new behaviours emerge as a consequence of those interactions and in response to the constantly changing nature of the problem? The surgeon-oncologist interaction depicted in the story might result in the surgeon implementing workarounds next time he faces a similar situation with the same oncologist.

Daily clinical problems are dynamic challenges defined multiply by individuals distributed across the system. Adopting an SSE lens can help us conceptualize a ‘problem’ not as singular and static, but as an entity that evolves, adapts and emerges across interactions and across time. Consistent with other recent innovations in medical education [4], we suggest that embracing a focus on the complexities of problem definition can enable novel educational practices that will equip trainees to effectively manage the problems they are likely to encounter in complex, team-based healthcare.
